# Dose‐Dependent Effects of Dietary n‐3 Fatty Acids on Bowel Health: Plant‐Sourced ALA Modulates Diarrhea Risk While Marine‐Sourced DHA/EPA Prevent Constipation in NHANES 2005–2010

**DOI:** 10.1002/fsn3.70769

**Published:** 2025-08-06

**Authors:** Tingting Li, Yihui Shi, Lijun Cai

**Affiliations:** ^1^ The First School of Clinical Medicine Zhejiang Chinese Medical University Hangzhou China; ^2^ Department of Gastroenterology The First Affiliated Hospital of Zhejiang Chinese Medical University (Zhejiang Provincial Hospital of Chinese Medicine) Hangzhou China

**Keywords:** constipation, diarrhea, NHANES, omega‐3 polyunsaturated fatty acid, weighted logistic regression

## Abstract

Although omega‐3 polyunsaturated fatty acid (n‐3PUFA) is generally considered to have positive effects on bowel health, the understanding of the impacts of n‐3PUFA on bowel function is limited. This study aimed to deeply investigate the association between n‐3PUFA, diarrhea, and constipation and discussed the role of different subclasses of n‐3PUFA. 12,704 adults from the National Health and Nutrition Examination Survey (NHANES) 2005–2010 were collected in this study. Constipation and diarrhea were measured by bowel frequency and the Bristol Stool Form Scale (BSFS). Dietary n‐3PUFA intake was collected by 24‐h dietary interviews. Weighted logistic regression results showed that the highest quartile of n‐3PUFA intake (OR: 0.70; 95% CI: 0.52–0.96) was associated with a reduced risk of constipation, and the third quartile of n‐3PUFA (OR: 0.70; 95% CI: 0.56–0.87) was linked to a lower risk of diarrhea (compared to the lowest). According to restricted cubic spline (RCS) analysis, n‐3PUFA showed a nonlinear association with diarrhea and a negative linear correlation with constipation. Moderate intake of n‐3PUFA (1.38–2.25 g/day) was associated with a reduced risk of diarrhea. When the intake of n‐3PUFA was more than 2.25 g per day, the risk of diarrhea increased. RCS‐adjusted logistic regression indicated that the highest ALA quartile (vs. lowest) was associated with 31% lower odds of diarrhea (OR: 0.69; 95% CI: 0.57–0.85) with a U‐shaped relationship. Conversely, higher DHA (OR: 0.60; 95% CI: 0.43–0.84) and EPA (OR: 0.70; 95% CI: 0.52–0.95) quartiles reduced constipation odds, showing negative linear (DHA) and nonlinear (EPA) associations. WQS regression quantified joint effects of n‐3PUFA subclasses on constipation/diarrhea. For diarrhea, n‐3PUFA contributions were: ALA 82.9%, EPA 16.5%, DHA 0.5%, DPA 0.1%; for constipation: DHA 75.8%, EPA 20.2%, ALA 3.0%, DPA 1.0%. Sensitivity analyses confirmed robust n‐3PUFA and subclass associations with diarrhea/constipation. This large‐scale study establishes differential impacts of n‐3PUFA subclasses on bowel disorders, revealing ALA's U‐shaped protection against diarrhea and DHA/EPA's linear constipation alleviation, providing evidence for targeted dietary interventions. However, the causal relationship still needs to be verified by prospective studies.

## Graphical abstract

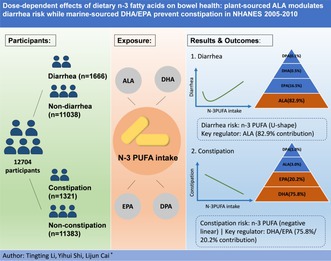

**Abbreviations**
ALA: alpha‐linolenic acid
BSFS: Bristol Stool Form Scale
CI: confidence interval
DHA: docosahexaenoic acid
DPA: docosapentaenoic acid
EPA: eicosapentaenoic acid
N‐3PUFA: omega‐3 polyunsaturated fatty acid
NHANES: National Health and Nutrition Examination Survey
OR: odds ratio
RCS: restricted cubic spline
WQS: weighted quantile sum

## 1 Introduction

Omega‐3 polyunsaturated fatty acid (n‐3PUFA), mainly from deep‐sea fish or fish oil supplements (Cholewski et al. 2018), is dietary fat with numerous cardiovascular, neurological, cognitive, and immune health benefits (Bahadorpour et al. 2023; Calder and Yaqoob 2009; Farley et al. 2021; Sasaki et al. 2024). It has been reported to promote bowel health by increasing the number of beneficial gut bacteria such as Bifidobacteria and Lactobacillus (Chai et al. 2024; Parolini 2019). N‐3PUFA also helps with intestinal diseases such as inflammatory bowel disease (IBD) (Ferguson et al. 2010), irritable bowel syndrome (Linsalata et al. 2024), and colon cancer (Cockbain et al. 2012). As diarrhea is a common symptom of IBD, this would seem to support that n‐3PUFA is beneficial for diarrhea. However, some studies have pointed out that n‐3PUFA can aggravate IBD (Schwärzler et al. 2022; Wen et al. 2023) and bring adverse effects such as diarrhea and nausea (Li et al. 2022). Moreover, n‐3PUFA is traditionally thought to have a positive effect on constipation, but studies have shown that n‐3PUFA prescription medications may have constipation side effects (Chinese Medical Association Geriatrics 2024). The results are controversial, and no studies have illustrated a direct and exact association between n‐3PUFA and bowel function.

Constipation and diarrhea are the main functional gastrointestinal disorders. According to statistics, 17% of Americans suffer from constipation (Choung et al. 2007), and the chances of having diarrhea are as high as 20% to 30% (Zhao et al. 2012). They are mainly characterized by changes in bowel habits and stool shape. In mild cases, it affects life and work, and in severe cases, it leads to complications such as dystrophy, hemorrhoids, and colorectal cancer (Diarrhoea and Malnutrition 1991; Fox et al. 2014; Wu et al. 2023), which negatively affect human health and bring mental and economic stress (Ballou et al. 2019; Sommers et al. 2015). Since diet is a significant factor influencing constipation and diarrhea, making dietary changes could be a more gentle and socially acceptable method to prevent the severe effects of diarrhea and constipation.

Our work is the first large‐scale population‐based study on n‐3PUFA and bowel function. By analyzing larger cross‐sectional data, this study provides information about the management and prevention of constipation and diarrhea from a dietary perspective.

## 2 Materials and Methods

### 2.1 Study Population

**FIGURE 1:**
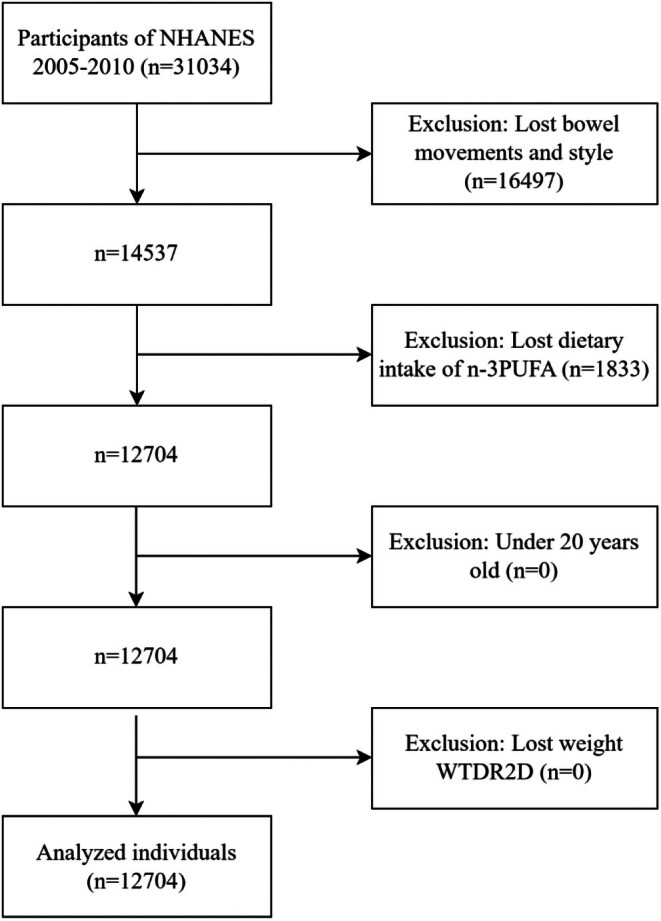
Participants' inclusion and exclusion processes.

Participant data were obtained from the National Health and Nutrition Examination Survey (NHANES), which incorporated information covering demographic, socioeconomic, dietary, and health issues to assess the health and nutritional status of the U.S. population. NHANES implemented a multi‐stage, unequal probability‐based selection design and provided sample weights to extrapolate the results. A total of 31,034 participants were included in NHANES 2005–2010, excluding the following groups: (1) age < 20 years; (2) loss of weight data; (3) did not complete the bowel health questionnaire; (4) Dietary n‐3PUFA intake data were missing. Eventually, 12,704 participants were included in the study (Figure 1).

NHANES is operated by the National Center for Health Statistics (NCHS) under the Centers for Disease Control (CDC) in the United States. All data collected from NHANES were approved by the NCHS Ethics Review Board (available on the website: https://www.cdc.gov/nchs/nhanes/), and all participants had written informed consent.

### 2.2 Assessment of Chronic Diarrhea and Constipation

Study participants were considered to have chronic diarrhea or constipation based on their responses to the Bowel Health Questionnaire. The questionnaire was completed through the Computer‐Assisted Individual Interview (CAPI) system in the interview room of the Mobile Medical Examination Center (MEC). Participants were presented with a color picture and the Seven Bristol Stool Shape Scale (BSFS; Type 1–7) and asked “Please look at this card and tell me the numbers that correspond to your usual or most common type of stool.” Participants are considered to have chronic constipation if they report that the most common stool type is type 1 (like nuts) or type 2 (sausage‐like, but lumpy). Participants with chronic diarrhea are classified as either type 6 (fluffy, ragged‐edged, mushy stool) or type 7 (watery, no solid pieces). Weekly bowel frequency is also used to measure bowel function and ask the following questions: “How many bowel movements do you typically have per week?” Based on previous NHANES data, less than 3 bowel movements per week are considered constipation, and more than 21 bowel movements per week are considered diarrhea. These measurements have been shown to be valid and have been widely used in other NHANES publications (Adejumo et al. 2019; Ballou et al. 2019; Liu et al. 2022, 2023; Peng et al. 2022; Singh et al. 2018). Individuals who do not have constipation and diarrhea are considered normal.

### 2.3 Assessment of Omega‐3 Polyunsaturated Fatty Acid Intake

Omega‐3 polyunsaturated fatty acid (n‐3PUFA) includes alpha‐linolenic acid (ALA), docosahexaenoic acid (DHA), eicosapentaenoic acid (EPA), and docosapentaenoic acid (DPA). In the present study, the intake of the four subtypes of n‐3PUFA was collected by two 24‐h dietary recalls. The first 24‐h food recall interview took place in a private room at the NHANES Mobile Health Center (MEC), where a set of measurement guides (measuring cups, spoons, rulers, bean bags, etc.) was provided. Participants were asked about the type and amount of food and drink consumed in the past 24 h. Second dietary recalls were collected after 3–10 days by telephone interview. The average of two 24‐h dietary recalls was taken to describe the daily intakes of ALA, DHA, EPA, and DPA. The Food and Nutrient Database for Dietary Studies (FNDDS) details the daily dietary energy and 64 nutrients in all foods, calculating the daily intake for a variety of food components, including ALA, DHA, EPA, and DPA. The daily intake of total n‐3PUFAs is the sum of the intakes of the four subtypes, which has been widely used in previous studies. It should be noted that the intake of n‐3PUFAs in this study includes dietary intake only, not supplemental n‐3PUFAs. Dietary supplements such as fish oil capsules were excluded from the analysis, and they were recorded separately in the Dietary Supplement Questionnaire (DSQ) of NHANES. Intake levels were expressed through quartiles. The lowest quartile (Q1) was used as a reference; Q2 and Q3 represent the 25%–50% and 50%–75% of n‐3PUFA intake in the population, respectively, and Q4 represents the highest level of intake.

### 2.4 Covariates

Variables that were or could be related to bowel function were included (Heitmann et al. 2021). Age, gender, race, education, and PIR (income‐to‐poverty ratio) were all included. Smoke status was divided into recent smoker, former smoker, and never smoker. Alcohol status was divided into drinking and non‐drinking. Besides, exercise, comorbidities, depression, and dietary habits were also included. Comorbidities include thyroid disease, coronary artery disease, high blood pressure, lung problems (asthma, emphysema or chronic bronchitis), chronic liver disease, chronic heart failure, stroke, and cancer. It has been shown that fiber, water intake, fat, caffeine, total sugar, and total energy intake are closely related to intestinal function (Heitmann et al. 2021), so these six categories were included and categorized in the analysis, as used in other studies (Markland et al. 2013). Dietary ingredient intake was collected through two 24‐h dietary interviews with NHANES. Explanation and classification of covariates were provided in Table S1.

### 2.5 Statistical Analysis

Firstly, the baseline characteristics of the population were analyzed, in which categorical variables were assessed using frequency counts and weighted posterior odds ratios. Model 1 is a crude model containing only n‐3 PUFA intake and outcome variables. Model 2 is based on the crude model by adding age analysis. Model 3 included n‐3 PUFA, demographics, lifestyle, comorbidities, and dietary habits. Confidence intervals (CIs) were all 95% CIs. Results were considered statistically significant if the 95% CIs did not contain 1. Then, restricted cubic spline (RCS) regression models were developed to evaluate the linear or nonlinear relationship between n‐3 PUFA and bowel function. Finally, the mixed effects of various types of n‐3 PUFA on constipation and diarrhea were analyzed by the weighted quantile sum (QWS) models, and the contribution of each n‐3 PUFA to reducing the odds of constipation and diarrhea was obtained. All statistics were implemented by R software (Version 4.4.1). Given the complexity of sampling‐based designs in NHANES, we added weights to extrapolate the findings to the US population. Because of the possible difference in dietary intake between weekdays and weekends during the week, we chose 24‐h dietary recall weights wtdrd2 to adjust for this difference, and this choice of weights is also recommended by NHANES.

## 3 Results

### 3.1 Participant Characteristics

The participants' baseline characteristics were displayed in Table 1, and those with a high intake of n‐3PUFA were more likely to be white, male, aged 50–59 years, with an alcohol use history, and overweight/obese. The percentage of individuals with diarrhea decreased (weighted) as the total n‐3PUFA intake increased, but it increased significantly when the daily intake exceeded almost 2 g (Q1: 25.0% vs. Q2: 21.4% vs. Q3: 19.9% vs. Q4: 33.7%), as shown in Table 2. This association was also found in the four subclasses. Additionally, constipation rates declined as total n‐3PUFA intake rises (Q1: 31.6% vs. Q2: 28.6% vs. Q3: 21.8% vs. Q4: 18.0%). Similarly, n‐3PUFA subclasses demonstrated comparable associations with constipation.

**TABLE 1:** Baseline characteristics of the study participants by quintile (Q) of n‐3PUFA intake.

Characteristic	Total n‐3PUFA intake				
	Q1^a^ (*n* = 3177)	Q2^a^ (*n* = 3175)	Q3^a^ (*n* = 3176)	Q4^a^ (*n* = 3176)	*p*
Age (%)					< 0.001
20–29	523 (20.0%)	501 (18.4%)	559 (18.3%)	547 (17.6%)	
30–39	446 (16.6%)	485 (16.6%)	551 (20.1%)	588 (20.3%)	
40–49	491 (18.7%)	555 (21.3%)	553 (19.8%)	593 (20.3%)	
50–59	463 (17.1%)	459 (16.3%)	502 (18.6%)	552 (21.9%)	
60–69	559 (13.0%)	574 (14.5%)	470 (12.3%)	493 (12.1%)	
≥ 70	695 (14.6%)	601 (12.9%)	541 (11.0%)	403 (7.9%)	
Gender (%)					< 0.001
Female	2077 (66.9%)	1781 (58.0%)	1539 (50.0%)	1191 (37.8%)	
Race (%)					0.003
Hispanic	964 (14.1%)	873 (13.5%)	764 (11.5%)	689 (10.3%)	
Non‐Hispanic black	620 (12.0%)	589 (11.3%)	610 (10.5%)	651 (10.4%)	
Non‐Hispanic white	1499 (69.6%)	1605 (70.8%)	1671 (72.3%)	1692 (73.4%)	
Other	94 (4.3%)	108 (4.4%)	131 (5.7%)	144 (5.9%)	
Education (%)					< 0.001
Below college	1922 (50.7%)	1631 (43.3%)	1523 (38.6%)	1381 (35.7%)	
PIR (%)					< 0.001
Below 2	1596 (39.0%)	1379 (32.8%)	1202 (28.0%)	1123 (24.5%)	
Smoke (%)					< 0.001
Former smoker	768 (22.8%)	821 (24.4%)	852 (25.3%)	887 (27.8%)	
Never smoker	1697 (51.4%)	1713 (55.1%)	1676 (53.8%)	1624 (52.7%)	
Recent smoker	712 (25.7%)	641 (20.5%)	648 (21.0%)	665 (19.5%)	
Drinker (%)	2074 (72.1%)	2171 (71.7%)	2286 (76.3%)	2516 (82.5%)	< 0.001
Regular vigorous activity (%)	803 (31.3%)	948 (34.6%)	1097 (39.8%)	1246 (44.7%)	< 0.001
Diabetes (%)	635 (13.8%)	518 (12.0%)	480 (10.9%)	405 (9.1%)	< 0.001
BMI (%)					0.583
Normal	901 (32.0%)	862 (30.0%)	935 (31.6%)	939 (30.6%)	
Obesity	1223 (35.9%)	1225 (36.2%)	1144 (34.8%)	1129 (34.0%)	
Overweight	1053 (32.1%)	1088 (32.8%)	1097 (33.6%)	1108 (35.4%)	
Comorbidities (%)					< 0.001
0	1283 (43.9%)	1440 (48.7%)	1571 (52.5%)	1558 (51.1%)	
1	1056 (32.5%)	983 (30.5%)	938 (29.8%)	985 (31.4%)	
2	562 (15.7%)	496 (13.8%)	434 (11.9%)	442 (13.2%)	
3	181 (5.1%)	173 (4.7%)	68 (4.1%)	138 (3.1%)	
4 and more	95 (2.7%)	83 (2.2%)	65 (1.7%)	53 (1.1%)	
Depression (%)	360 (10.7%)	277 (8.0%)	233 (6.1%)	217 (5.9%)	< 0.001
Highest intake of fiber^b^ (%)	291 (9.1%)	595 (18.0%)	920 (27.8%)	1367 (45.0%)	< 0.001
Highest intake of water^b^ (%)	719 (26.0%)	761 (26.7%)	750 (25.7%)	943 (33.3%)	< 0.001
Highest intake of fat^b^ (%)	35 (1.8%)	275 (9.9%)	892 (29.3%)	1974 (64.0%)	< 0.001
Highest intake of caffeine^b^ (%)	599 (25.0%)	714 (26.7%)	898 (33.3%)	965 (36.4%)	< 0.001
Highest intake of energy^b^ (%)	80 (3.3%)	337 (11.5%)	922 (29.5%)	1837 (59.8%)	< 0.001
Highest intake of sugar^b^ (%)	353 (12.3%)	611 (19.9%)	926 (28.0%)	1286 (40.1%)	< 0.001

^a^ Q1 is the first quartile of omega‐3 fatty acid intake (consuming less than 0.9385 g of n‐3PUFA per day), Q2 is the second quartile of omega‐3 fatty acid intake (consuming 0.9385–1.3823 g/day), Q3 is the third quartile of omega‐3 fatty acid intake (consuming 1.3823–1.9866 g/day), and Q4 is the highest quartile of omega‐3 fatty acid intake (consuming more than 1.9816 g/day).

^b^ All dietary factors were measured using a 24‐h dietary recall interview that included detailed questions about over 60 foods and nutrients consumed during the previous 24‐h period. The average of two interviews, spaced three to ten days apart, was used to calculate the daily nutrient intake (provided in Table S2).

**TABLE 2:** Intake of n‐3 PUFA in patients with diarrhea and constipation.

	Diarrhea		*p*	Constipation		*p*
	No (11038)	Yes (1666)		No (11383)	Yes (1321)	
Total n‐3PUFA (gm per day)			**< 0.001**			**< 0.001**
Q1 (0.0000–0.9385)	2720 (22.9%)	457 (25.0%)		2747 (22.2%)	430 (31.6%)	
Q2 (0.9385–1.3823)	2783 (24.1%)	392 (21.4%)		2825 (23.2%)	350 (28.6%)	
Q3 (1.3823–1.9816)	2806 (25.8%)	370 (19.9%)		2868 (25.4%)	308 (21.8%)	
Q4 (1.9816–18.3695)	2729 (27.3%)	447 (33.7%)		2943 (29.1%)	233 (18.0%)	
ALA (gm per day)			**0.002**			**< 0.001**
Q1 (0.0000–0.8625)	2727 (22.4%)	449 (25.0%)		2750 (21.9%)	426 (29.9%)	
Q2 (0.8625–1.2723)	2766 (24.3%)	409 (22.3%)		2825 (23.6%)	350 (28.1%)	
Q3 (1.2723–1.8110)	2797 (25.8%)	380 (20.7%)		2883 (25.5%)	294 (22.4%)	
Q4 (1.8100–18.3400)	2748 (27.5%)	428 (32.0%)		2925 (29.0%)	251 (19.6%)	
DHA (gm per day)			**0.012**			**< 0.001**
Q1 (0.0000–0.0105)	2753 (25.6%)	387 (24.1%)		2769 (24.7%)	371 (31.8%)	
Q2 (0.0105–0.0305)	2770 (25.3%)	396 (21.0%)		2809 (24.5%)	357 (27.1%)	
Q3 (0.0305–0.0720)	2778 (24.4%)	420 (24.6%)		2863 (24.4%)	335 (24.0%)	
Q4 (0.0720–2.8940)	2737 (24.8%)	463 (30.3%)		2942 (26.3%)	258 (17.1%)	
EPA (gm per day)			0.081			**0.001**
Q1 (0.0000–0.0035)	2723 (24.2%)	410 (23.8%)		2769 (23.5%)	364 (30.0%)	
Q2 (0.0035–0.0085)	2785 (24.9%)	405 (21.7%)		2838 (24.4%)	352 (26.1%)	
Q3 (0.0085–0.0225)	2785 (25.6%)	407 (25.6%)		2858 (25.6%)	334 (25.5%)	
Q4 (0.0225–1.9910)	2745 (25.2%)	444 (28.8%)		2918 (26.3%)	271 (18.4%)	
DPA (gm per day)			**0.049**			**0.001**
Q1 (0.0000–0.0050)	2678 (24.7%)	382 (24.9%)		2700 (24.3%)	360 (28.8%)	
Q2 (0.0050–0.0130)	2805 (25.7%)	402 (21.8%)		2847 (24.9%)	360 (28.7%)	
Q3 (0.0130–0.0255)	2814 (25.5%)	437 (25.4%)		2935 (25.7%)	316 (23.3%)	
Q4 (0.0255–0.4810)	2741 (24.1%)	445 (28.0%)		2901 (25.2%)	285 (19.2%)	

*Note:* Bold values indicate statistically significant results (*p* < 0.05).

### 3.2 Association Between n‐3PUFA Intake and Diarrhea

Each variable's relationship to diarrhea was assessed using univariate linear regression; variables with *p* < 0.1 were included in the regression model, while those with *p* > 0.1 were thought to be less related to diarrhea (shown in Table S2). Eventually, alcohol consumption, caffeine intake, and sugar intake were excluded.

According to the results of weighted logistic regression, a lower risk of diarrhea was linked to total n‐3PUFA. As shown in Table 3, the third quartile of n‐3PUFA was associated with a 29%, 30%, and 30% reduction in the risk of diarrhea in models 1, 2, and 3 (OR [95% CI]: Model 1: 0.71 [0.59, 0.85]; Model 2: 0.70 [0.58, 0.85]; Model 3: 0.70 [0.56, 0.87]).

**TABLE 3:** Weighted logistic regression models of total n‐3 PUFA intake and diarrhea.

	OR 95% CI			
	Q1 (0–0.9435 g)	Q2 (0.9435–1.3893 g)	Q3 (1.3893–1.9851 g)	Q4 (1.9851–18.3695 g)
Model 1	Reference	0.81 [0.64, 1.02]	**0.71 [0.59, 0.85]**	1.13 [0.93, 1.37]
*p*		0.074	**0.001**	0.209
Model 2	Reference	0.80 [0.64,1.01]	**0.70 [0.58, 0.85]**	1.12 [0.92, 1.37]
*p*		0.060	**0.001**	0.236
Model 3	Reference	0.81 [0.64, 1.04]	**0.70 [0.56, 0.87]**	1.01 [0.78, 1.31]
*p*		0.091	**0.003**	0.955

*Note:* Model 1, The crude model; Model 2, The age‐adjusted model; Model 3, Demographic factors, lifestyle, eating habits, and comorbidities were adjusted in this model. Bold values indicate statistically significant results (*p* < 0.05% and 95% confidence interval does not include 1).

As indicated in Table 4, the results of logistic regression demonstrated that ALA was linked to a lower incidence of diarrhea, whereas DHA, EPA, and DPA were not significantly linked to diarrhea. There was a 28%, 29%, and 30% decrease in the risk of diarrhea in Model 1, Model 2, and Model 3, respectively, when ALA was in the third quartile. To reduce possible interference with the results from interactions between n‐3 PUFAs, we included n‐3 PUFAs in Model 4 in addition to study subjects and found that ALA was still significantly associated with diarrhea (OR: 0.70; 95% CI: 0.51–0.96).

**TABLE 4:** Weighted logistic regression models of four n‐3 PUFA subclasses and diarrhea.

	Diarrhea (95% CI)							
	Model 1	*p*	Model 2	*p*	Model 3	*p*	Model 4	*p*
ALA (g per day)								
Q1 (0.0000–0.8639)	Reference		Reference		Reference		Reference	
Q2 (0.8639–1.2720)	0.83 [0.66, 1.03]	0.090	0.81 [0.65, 1.01]	0.067	0.83 [0.67, 1.04]	0.099	0.83 [0.67, 1.03]	0.091
Q3 (1.2720–1.8126)	**0.72 [0.60, 0.86]**	**0.001**	**0.71 [0.59, 0.86]**	**0.001**	**0.70 [0.57, 0.85]**	**0.001**	**0.69 [0.57, 0.85]**	**0.001**
Q4 (1.8126–18.3370)	1.04 [0.87, 1.25]	0.644	1.04 [0.87, 1.24]	0.683	0.87 [0.71, 1.07]	0.174	0.86 [0.71, 1.04]	0.119
DHA (g per day)								
Q1 (0.0000–0.0110)	Reference		Reference		Reference		Reference	
Q2 (0.0110–0.0310)	0.88 [0.72, 1.07]	0.198	0.86 [0.71, 1.06]	0.151	0.86 [0.70, 1.05]	0.138	0.86 [0.70, 1.05]	0.136
Q3 (0.0310–0.0735)	1.07 [0.87, 1.31]	0.507	1.05 [0.85, 1.30]	0.625	1.02 [0.81, 1.27]	0.871	1.02 [0.81, 1.28]	0.894
Q4 (0.0735–3.2025)	**1.29 [1.01, 1.67]**	**0.046**	1.27 [0.98, 1.64]	0.074	1.22 [0.91, 1.63]	0.170	1.21 [0.91, 1.61]	0.168
EPA (g per day)								
Q1 (0.0000–0.0035)	Reference		Reference		Reference		Reference	
Q2 (0.0035–0.0085)	0.89 [0.74, 1.06]	0.196	0.89 [0.74, 1.06]	0.183	0.88 [0.74, 1.05]	0.158	0.88 [0.74, 1.04]	0.132
Q3 (0.0085–0.0235)	1.02 [0.85, 1.23]	0.821	1.02 [0.84, 1.24]	0.851	0.98 [0.79, 1.22]	0.852	0.97 [0.78, 1.20]	0.751
Q4 (0.0235–1.9910)	1.16 [0.92, 1.47]	0.201	1.15 [0.91, 1.47]	0.234	1.14 [0.88, 1.48]	0.317	1.10 [0.88, 1.38]	0.365
DPA (g per day)								
Q1 (0.0000–0.0040)	Reference		Reference		Reference		Reference	
Q2 (0.0040–0.0120)	0.84 [0.69, 1.03]	0.097	0.85 [0.69, 1.04]	0.102	0.84 [0.69, 1.03]	0.091	0.84 [0.68, 1.03]	0.083
Q3 (0.0120–0.0250)	0.99 [0.79, 1.25]	0.936	0.99 [0.79, 1.26]	0.964	0.97 [0.77, 1.23]	0.800	0.96 [0.76, 1.22]	0.718
Q4 (0.0250–1.1015)	1.15 [0.90, 1.48]	0.265	1.14 [0.88, 1.48]	0.302	1.06 [0.79, 1.43]	0.691	1.01 [0.77, 1.34]	0.924

*Note:* Bold values indicate statistically significant results (*p* < 0.05% and 95% confidence interval does not include 1).

**FIGURE 2:**
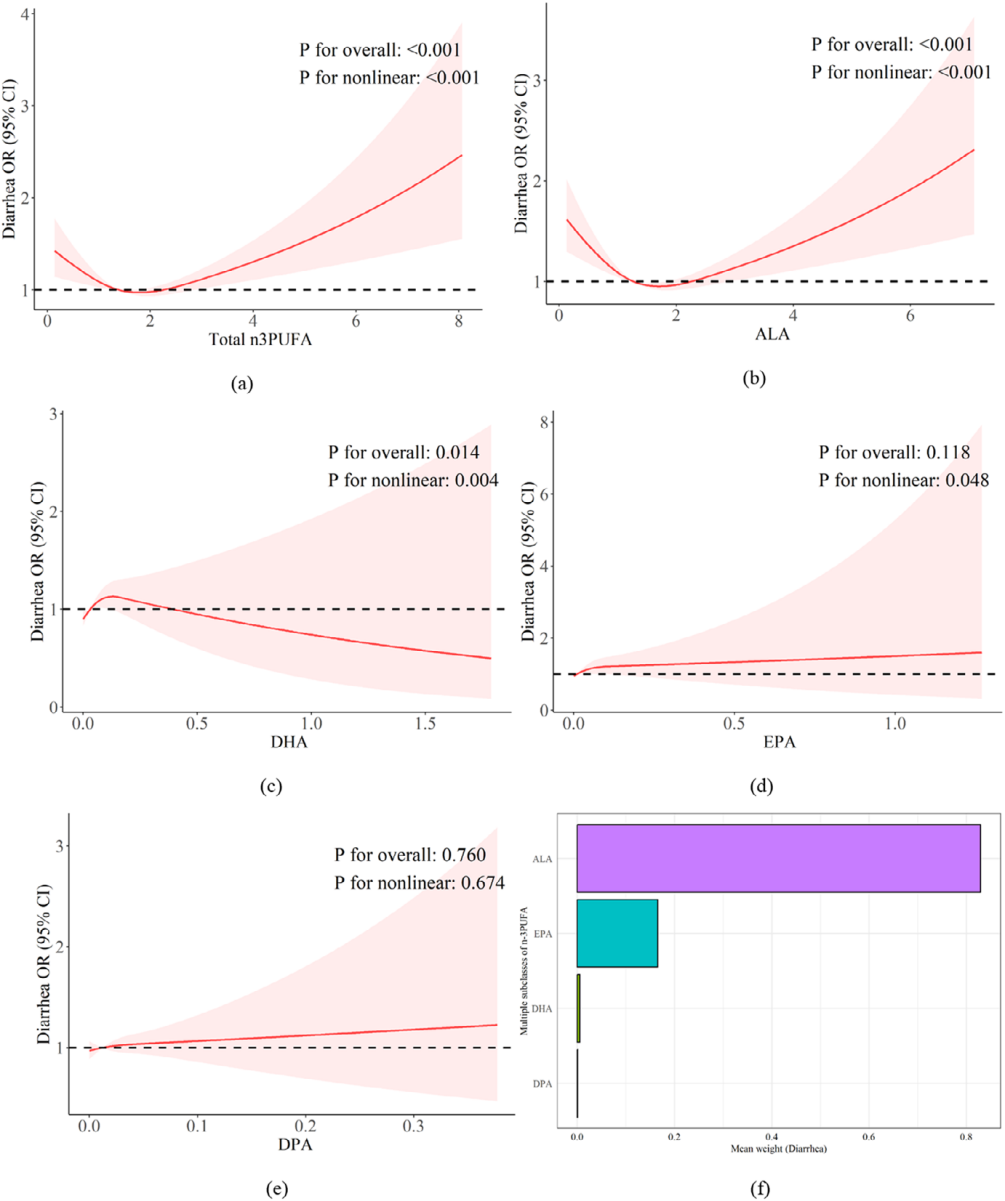
Dose‐relationship and the WQS results between n‐3 PUFAs and diarrhea. (a–e) Dose relationship between diarrhea and total n‐3 PUFA (a), ALA (b), DHA (c), EPA (d), and DPA (e). (f) Combined effects of n‐3UFA subclasses on diarrhea. n‐3 PUFA contributions were: ALA 82.9%, EPA 16.5%, DHA 0.5%, DPA 0.1%.

As shown in Figure 2a, there was a significant nonlinear association between n‐3 PUFA intake and diarrhea (*p* < 0.001). The tangent points are at *x* = 1.38 and *x* = 2.25. The risk of diarrhea is decreased when n‐3 PUFA intake is between 1.38 and 2.25 g per day; on the other hand, the risk of diarrhea is increased when n‐3 PUFA intake surpasses 2.25 g per day. Given that there were 2.3 g of n‐3 PUFA for every 100 g of salmon, consuming roughly 97.8 g of salmon daily was associated with increased odds of diarrhea. Additionally, the RCS findings showed a nonlinear relationship between ALA (Figure 2b), DHA (Figure 2c), and EPA (Figure 2d) and a linear relationship between DPA (Figure 2e) and diarrhea.

As shown in Figure 2f, the WQS results showed the contribution of each subset of n‐3 PUFA to diarrhea. ALA was a significant contributor to reduced odds of diarrhea (weight: 82.9%), followed by EPA (weight: 16.5%), DHA (weight: 0.5%), and DPA (weight: 0.1%).

### 3.3 Association Between n‐3PUFA Intake and Constipation

**FIGURE 3:**
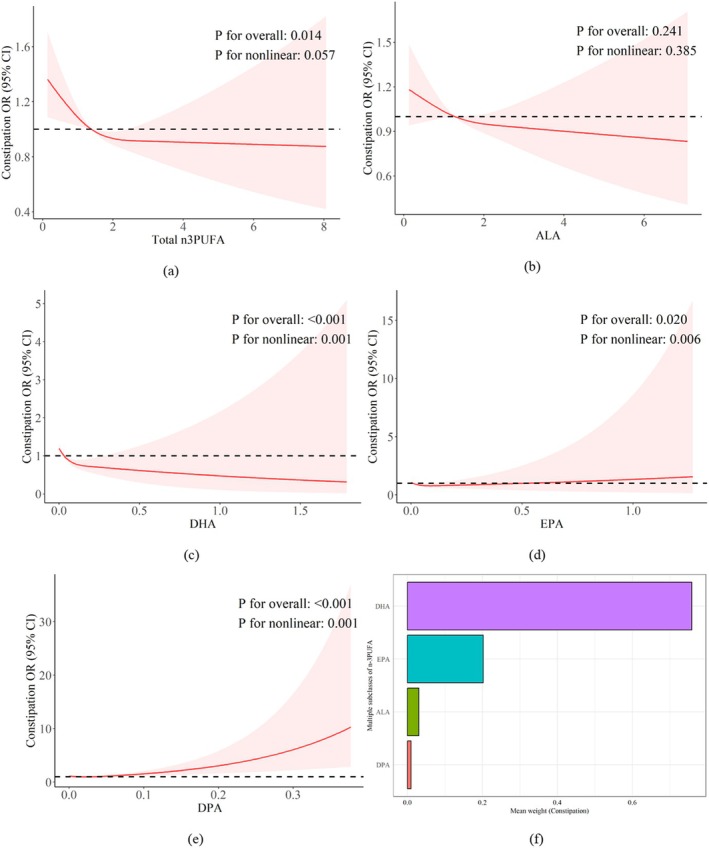
Dose‐relationship and the WQS results between n‐3PUFAs and constipation. (a–e) Dose relationship between constipation and total n‐3PUFA (a), ALA (b), DHA (c), EPA (d), and DPA (e). (f) Combined effects of n‐3UFA subclasses on constipation. n‐3PUFA contributions were: DHA 75.8%, EPA 20.2%, ALA 3.0%, DPA 1.0%.

Univariate linear regression (provided in Table S2) excluded variables that were less associated with constipation (*p* > 0.1): comorbidities and sugar intake. As shown in Table 5, the highest quartile of total n‐3PUFA intake was significantly associated with reduced constipation risk (OR: 0.70; 95% CI: 0.52–0.96) after full covariate adjustment. Table 6 demonstrates that the highest quartiles of DHA and EPA were associated with 40% and 30% lower constipation odds, respectively (DHA: OR 0.60, 95% CI: 0.43–0.84; EPA: OR 0.70, 95% CI: 0.52–0.95). RCS analysis revealed a significant negative linear association between total n‐3PUFA and constipation, while DHA, EPA, and DPA showed nonlinear associations. ALA exhibited a nonsignificant linear relationship. WQS results further indicated DHA (75.8%) and EPA (20.2%) as primary contributors to the joint effect of n‐3PUFA on constipation, with minimal contributions from ALA (3.0%) and DPA (1.0%) (Figure 3).

**TABLE 5:** Weighted logistic regression models of total n‐3 PUFA intake and constipation.

	OR 95% CI			
	Q1 (0–0.9435 g)	Q2 (0.9435–1.3893 g)	Q3 (1.3893–1.9851 g)	Q4 (1.9851–18.3695 g)
Model 1	Reference	0.87 [0.70, 1.07]	**0.60 [0.49, 0.75]**	**0.43 [0.34, 0.55]**
*p*		0.170	**< 0.001**	**< 0.001**
Model 2	Reference	0.60 [0.49, 0.75]	**0.60 [0.48, 0.75]**	**0.44 [0.34, 0.56]**
*p*		0.201	**< 0.001**	**< 0.001**
Model 3	Reference	1.01 [0.82, 1.25]	0.79 [0.62, 1.00]	**0.70 [0.52, 0.96]**
*p*		0.902	0.053	**0.027**

*Note:* Model 1, The crude model; Model 2, The age‐adjusted model; Model 3, Demographic factors, lifestyle, eating habits and comorbidities were adjusted in this model. Bold values indicate statistically significant results (*p* < 0.05% and 95% confidence interval does not include 1).

**TABLE 6:** Weighted logistic regression models of four n‐3PUFA subclasses and constipation.

	Constipation (95% CI)							
	Model 1	*p*	Model 2	*p*	Model 3	*p*	Model4	*p*
ALA (g per day)								
Q1 (0.0000–0.8639)	Reference		Reference		Reference		Reference	
Q2 (0.8639–1.2720)	0.87 [0.71, 1.07]	0.191	0.89 [0.72, 1.08]	0.224	1.04 [0.85, 1.27]	0.700	1.05 [0.85, 1.29]	0.634
Q3 (1.2720–1.8126)	**0.64 [0.50, 0.82]**	**0.001**	**0.65 [0.51, 0.83]**	**0.001**	0.87 [0.66, 1.14]	0.296	0.88 [0.66, 1.16]	0.340
Q4 (1.8126–18.3370)	**0.50 [0.40, 0.63]**	**< 0.001**	**0.50 [0.40, 0.63]**	**< 0.001**	0.86 [0.65, 1.12]	0.249	0.87 [0.67, 1.15]	0.312
DHA (g per day)								
Q1 (0.0000–0.0110)	Reference		Reference		Reference		Reference	
Q2 (0.0110–0.0310)	0.86 [0.66, 1.11]	0.234	0.87 [0.67, 1.13]	0.293	0.88 [0.67, 1.14]	0.307	0.86 [0.66, 1.13]	0.275
Q3 (0.0310–0.0735)	0.76 [0.59, 0.98]	0.034	0.78 [0.60, 1.01]	0.054	0.89 [0.67, 1.17]	0.389	0.86 [0.64, 1.15]	0.292
Q4 (0.0735–3.2025)	**0.51 [0.39, 0.65]**	**< 0.001**	**0.52 [0.40, 0.67]**	**< 0.001**	**0.65 [0.50, 0.84]**	**0.002**	**0.60 [0.43, 0.84]**	**0.006**
EPA (g per day)								
Q1 (0.0000–0.0035)	Reference		Reference		Reference		Reference	
Q2 (0.0035–0.0085)	0.84 [0.66, 1.07]	0.155	0.84 [0.66, 1.07]	0.160	0.87 [0.68, 1.12]	0.267	0.86 [0.67, 1.11]	0.231
Q3 (0.0085–0.0235)	0.78 [0.59, 1.04]	0.090	0.79 [0.58, 1.06]	0.106	0.92 [0.68, 1.24]	0.568	0.89 [0.65, 1.23]	0.469
Q4 (0.0235–1.9910)	**0.54 [0.44, 0.67]**	**< 0.001**	**0.55 [0.45, 0.68]**	**< 0.001**	**0.70 [0.56, 0.88]**	**0.004**	**0.70 [0.52, 0.95]**	**0.024**
DPA (g per day)								
Q1 (0.0000–0.0040)	Reference		Reference		Reference		Reference	
Q2 (0.0040–0.0120)	0.97 [0.80, 1.19]	0.792	0.97 [0.80, 1.18]	0.765	1.00 [0.79, 1.25]	0.964	1.01 [0.81, 1.27]	0.922
Q3 (0.0120–0.0250)	**0.77 [0.63, 0.94]**	**0.010**	**0.76 [0.63, 0.93]**	**0.010**	0.83 [0.67, 1.03]	0.083	0.86 [0.68, 1.07]	0.166
Q4 (0.0250–1.1015)	**0.64 [0.52, 0.81]**	**< 0.001**	**0.65 [0.52, 0.82]**	**< 0.001**	0.86 [0.68, 1.09]	0.203	0.96 [0.73, 1.27]	0.761

*Note:* Bold values indicate statistically significant results (*p* < 0.05% and 95% confidence interval does not include 1).

### 3.4 Sensitive Analysis

To evaluate the robustness of our findings, we conducted a sensitivity analysis excluding participants with potential organic gastrointestinal conditions. Individuals with self‐reported inflammatory bowel disease (IBD) (*n* = 46), celiac disease (*n* = 9), and colorectal cancer (*n* = 92) were excluded. Individuals with both constipation and diarrhea (*n* = 77) were also excluded. Sensitivity analyses confirmed the robustness of associations between: (1) total n‐3 PUFA and ALA with diarrhea, and (2) total n‐3 PUFA and DHA with constipation. After exclusion, the association between higher intake of total n‐3 PUFA/ALA (Q3 vs. Q1) and reduced risk of chronic diarrhea remained statistically significant (total n3PUFA: OR = 0.71, 95% CI: 0.58–0.88; ALA: OR = 0.71, 95% CI: 0.57–0.89). Furthermore, the highest quartile of n‐3 PUFA and DHA intake remained significantly associated with reduced odds of constipation (total n‐3 PUFA: OR = 0.68, 95% CI: 0.50–0.92; DHA: OR = 0.62, 95% CI: 0.43–0.89). However, the association between EPA and constipation attenuated after full covariate adjustment. More on the results of the sensitivity analysis can be found in Tables S3, S4.

## 4 Discussion

Our study provided novel insights into the complex, subtype‐specific relationships between dietary n‐3 PUFA and bowel function (diarrhea and constipation). Analyzing 12,704 participants from NHANES 2005–2010, we demonstrated three key findings: Firstly, total n‐3 PUFA exhibited a nonlinear association with diarrhea and a significant inverse linear relationship with constipation. Secondly, ALA intake at the third quartile showed a nonlinear association with reduced diarrhea odds, while DHA and EPA at the highest quartile demonstrated nonlinear inverse associations with constipation risk. Thirdly, weighted contribution analysis revealed differential subtype dominance: ALA accounted for 82.9% of the combined anti‐diarrheal effect, whereas DHA (75.8%) and EPA (20.2%) collectively contributed 96.0% to constipation risk reduction.

Our results were partially consistent with previously published research. For example, a study of fish oil for colitis in mice showed that a diet supplemented with fish oil (containing DHA and EPA) improved diarrhea in mice (Sharma et al. 2019). In addition, studies of flaxseed oil by Hanif Palla and Gilani (2015) showed that oral administration of flaxseed oil (mainly containing ALA) reduced diarrhea scores; similarly, a study by Bartosz Malinowski et al. showed that fatty acid supplementation in patients with Crohn's disease and inflammatory bowel disease improved their quality of life and caused clinical relief of symptoms such as abdominal pain and diarrhea (Malinowski et al. 2020). However, these conclusions were mainly based on fish oil supplements or other oils that contained fatty acids. Studies of various n‐3 PUFAs from dietary intake sources alone were lacking. However, most existing studies relied on purified supplements, overlooking critical food matrix interactions. This approach failed to establish human‐relevant intake thresholds. Additionally, they largely neglected to investigate potential nonlinear dose–response dynamics, particularly the attenuation of benefits or risk elevation at higher intake levels.

The U‐shaped relationship between total n‐3 PUFA intake and diarrhea risk likely stemmed from complex dose‐dependent physiological interactions. Moderate consumption (1.38–2.25 g/day) was associated with reduced diarrhea incidence, whereas exceeding 2.25 g/day attenuated this protection. Previous studies demonstrated that n‐3 PUFA increased the abundance of beneficial bacteria like Bifidobacteria and Lactobacillus, which induced anti‐inflammatory responses (Kaliannan et al. 2015; Quin et al. 2020; Watson et al. 2018). Simultaneously, they reduced levels of inflammation‐associated microorganisms such as Bacillus and *Clostridium coccoides* , thereby promoting intestinal barrier integrity (Cao et al. 2019; Fu et al. 2021). n‐3 PUFA further alleviated intestinal inflammation through dual pathways: suppressing pro‐inflammatory cytokines (TNF‐α, IL‐1β, IFN‐γ, IL‐6) and enhancing short‐chain fatty acid (SCFA, mainly butyrate and acetate) production (Cao et al. 2019; Husson et al. 2016; Kang and Weylandt 2008; Kim et al. 2018). Butyrate strengthened gut barrier function through FFAR2 (GPR43) receptor signaling. This stimulated mucin secretion and anti‐inflammatory cytokine production, reducing intestinal inflammation (Cao et al. 2019; Hays et al. 2024; Morris et al. 2017). Thus, n‐3 PUFA protected against diarrhea primarily through immunomodulation and microbiome regulation. Paradoxically, excessive intake (> 2.25 g/day) may increase diarrhea risk, as evidenced by elevated odds ratios (OR) at 2.32 ± 1.73 g/day in prior studies (Chang et al. 2023). This reversal was potentially due to microbial dysbiosis induced by lipid overload, though research on high‐dose n‐3 PUFA enterotoxicity remained notably limited.

The differential impact of n‐3 PUFA subtypes on diarrhea risk was striking. Alpha‐linolenic acid (ALA) emerged as the predominant contributor (79.9%) to the combined effect of the four major n‐3 PUFAs (ALA, EPA, DPA, DHA) on diarrhea. Consistently, a study by Kangwan et al. (2021) showed that perilla seed oil, which contains ALA, was able to reduce the chance of diarrhea in mice with colitis. Yang et al. (2023) demonstrated significant positive correlations between α‐linolenic acid (ALA) and gut microbiota, including *Bacteroides*, *Bifidobacterium*, *Prevotella*, *Paraprevotella*, and *Marseilliibacter*. Oral ALA administration reduced pro‐inflammatory cytokine expression by inhibiting the MyD88/NF‐κB pathway, thereby mitigating intestinal inflammation and diarrhea. Complementing this, Rodrigues et al. (2023) established that ALA intake promoted an anti‐inflammatory microbial balance. However, in this study, no significant association was found between the daily intake of DHA and EPA and diarrhea. This may be because this study covers a variety of chronic diarrhea types, such as inflammatory diarrhea, functional diarrhea, dysbiotic diarrhea, etc.

Total n‐3 PUFA intake demonstrated a significant inverse linear relationship with constipation risk, primarily mediated by DHA and EPA. When the intake level was in the third quartile, these long‐chain PUFAs were associated with reduced constipation odds through distinct neuro‐immune‐microbiota pathways. Previous studies indicated that DHA and EPA enhanced the production of short‐chain fatty acids (SCFAs), including acetate and butyrate. The beneficial effects of butyrate on alleviating constipation were mediated through its dual action on colonic transit. Butyrate stimulated choline acetyltransferase‐positive (ChAT^+^) neurons via the monocarboxylate transporter 2 (MCT2), thereby strengthening neuromuscular signaling to improve gut motility (Soret et al. 2010). Besides, butyrate modulated intestinal secretion and motility by promoting serotonin (5‐hydroxytryptamine, 5‐HT) synthesis (Gershon and Tack 2007; McLean et al. 2007; Reigstad et al. 2015). This physiological framework gained clinical support from recent evidence showing that maternal DHA supplementation (400 mg/day) significantly reduced the constipation risk of infants at 6 months postpartum (Khandelwal et al. 2025). The consistency between observational and interventional data underscored the biological plausibility of n‐3 PUFA in constipation regulation. Additionally, in vitro evidence demonstrated that SCFAs stimulated contraction of longitudinal smooth muscle in feline colon, enhancing propulsive activity to ameliorate colonic dysmotility (Rondeau et al. 2003). Collectively, the concordance between observational and interventional data underscored the biological plausibility of n‐3 PUFA‐mediated constipation regulation.

Crucially, our findings highlighted the importance of dose and food source. Ignoring the identified nonlinearities—the U‐shaped curve for ALA and diarrhea, and the linear benefit of n‐3 PUFA (driven by DHA/EPA) on constipation—could lead to misinterpretations of n‐3PUFA's gut health effects. We established a defined therapeutic window: moderate total n‐3 PUFA intake (≤ 2.25 g/day) provided digestive benefits. Exceeding these limits, especially for ALA, elevated diarrhea risk. This is an important public health concern given the growing use of ALA‐rich plant oils. As people pursue a healthy body shape, satiating foods like chia seeds and flaxseeds are becoming increasingly popular, as they contain a high amount of n‐3 PUFAs. It was reported that 1 tablespoon (about 10 g) of whole flaxseeds contains 2.3 g of ALA. 1 tablespoon (about 10 g) of chia seeds contains 1.8 g of ALA. Additionally, 1 oz of walnuts (about 28 g, or about 7 walnuts) contains 2.6 g of ALA. Excessive consumption of these ALA‐rich foods can increase the risk of diarrhea. Therefore, a correct understanding of the relationship between omega‐3 fatty acids and diarrhea can help form a healthy diet.

In conclusion, dietary n‐3 PUFA influence gut health through distinct pathways: ALA exhibits a narrow beneficial range for diarrhea prevention mediated largely via gut microbiota and their metabolites, while DHA and EPA demonstrate a linear protective association against constipation, likely involving microbiota‐driven stimulation of gut motility and neural signaling. These insights highlight the necessity of considering n‐3 PUFA subtype, dietary source, and intake level for personalized nutritional strategies aimed at optimizing gastrointestinal well‐being.

There were several advantages to our research. Firstly, our study systematically analyzed the heterogeneous effects of n‐3 PUFA subtypes on chronic diarrhea and constipation for the first time. Although previous studies have reported the protective effect of total n‐3 PUFAs on bowel health (Costantini et al. 2017; Fu et al. 2021; Yan et al. 2023), the independent and combined effects of different subtypes were unclear. In this study, the contribution weights of each subtype were first quantified by WQS analysis, and it was found that plant‐derived ALA contributed 82.9% of the combined effect, suggesting the potential leading role of plant‐derived n‐3 PUFAs in the prevention of chronic diarrhea. This finding may provide a new direction for dietary interventions. Secondly, by the RCS model, we observed that the association of total n‐3 PUFAs and ALA with chronic diarrhea was a U‐shaped curve. These results suggest that blindly increasing n‐3 PUFA intake may be counterproductive, and it is necessary to formulate accurate recommended amounts based on individual metabolic capacity. In the sensitivity analysis, the protective effect of total n‐3 PUFA (OR = 0.73) remained stable after excluding confounding cases and adjusting multiple models, supporting its reliability as an independent protective factor. In contrast, the association of ALA was attenuated by the inclusion of other fatty acids, suggesting that the observed effects of ALA may be partially mediated or confounded by interactions with other fatty acids. This is a dimension that has rarely been explored in previous nutritional epidemiology studies.

However, there were limitations to our study. The Rome criteria for diagnosing functional constipation or diarrhea were not applied, as the NHANES dataset lacked specific questionnaire items such as symptom duration or the need for manual maneuvers to facilitate defecation. Additionally, self‐reported data are susceptible to recall bias. Furthermore, due to the cross‐sectional design, causal relationships cannot be established. Future prospective studies are warranted to further confirm the observed associations, particularly regarding dose–response relationships and causality.

## 5 Conclusion

This study revealed a nonlinear relationship between total n‐3 PUFA intake and diarrhea risk, where moderate consumption (1.38–2.25 g/day) reduced odds by 30%, but excess (> 2.25 g/day) negates benefits. ALA drove 82.9% of this effect, exhibiting U‐shaped protection peaking at 1.29–2.23 g/day. Conversely, marine EPA/DHA linearly alleviated constipation (OR = 0.60–0.70). These findings supported distinct dietary strategies: optimizing plant‐based ALA within thresholds while increasing marine PUFA intake for bowel health, pending causal verification.

## Author Contributions

**Tingting Li:** conceptualization (equal), data curation (equal), formal analysis (equal), methodology (equal), software (equal), writing – original draft (equal). **Yihui Shi:** formal analysis (equal), investigation (equal), methodology (equal), validation (equal), visualization (equal), writing – original draft (equal). **Lijun Cai:** conceptualization (equal), supervision (equal), writing – review and editing (supporting).

## Ethics Statement

The Centers for Disease Control (CDC)–approved NHANES program, to which all participants consented. NHANES–National Health and Nutrition Examination Survey Homepage.

## Conflicts of Interest

The authors declare no conflicts of interest.

## Supporting information


**Table S1:** Explanation and classification of covariates.
**Table S2:** Univariate linear regression results of constipation and diarrhea.
**Table S3:** Logistic regression results of total n‐3PUFA with constipation and diarrhea in sensitive analysis.
**Table S4:** Logistic regression results of n‐3PUFA subtypes with constipation and diarrhea in sensitive analysis.

## Data Availability

This study was carried out using publicly available data from NHANES at https://www.cdc.gov/nchs/nhanes/index.htm.
