# Supplementary material for: Dose‐Dependent Effects of Dietary n‐3 Fatty Acids on Bowel Health: Plant‐Sourced ALA Modulates Diarrhea Risk While Marine‐Sourced DHA/EPA Prevent Constipation in NHANES 2005–2010
Source: Food Sci Nutr. 2025 Aug 6;13(8):e70769. doi: 10.1002/fsn3.70769 (PMC12326190; doi:10.1002/fsn3.70769)
Supplement: Supplementary file 1 — Table S1: Explanation and classification of covariates. Table S2: Univariate linear regression results of constipation and diarrhea. Table S3: Logistic regression results of total n‐3PUFA with constipation and diarrhea in sensitive analysis. Table S4: Logistic regression results of n‐3PUFA subtypes with constipation and diarrhea in sensitive analysis. [file FSN3-13-e70769-s001.docx]

**Table S1: Explanation and classification of covariates**

|  | **Covariates** | **Explanation and classification** |
| --- | --- | --- |
| Demographics | Age | 20-29,30-39,40-49,50-59,60-69, >=70 |
|  | Gender | Female; Male |
|  | Race | Hispanic; Non-Hispanic Black; Non-Hispanic White; Other |
|  | Education | Below college, college and above |
|  | PIR | The ratio of family income to poverty. |
|  | BMI | Body mass index. Overweight is defined as a BMI<25kg/m^2^, obesity is defined as a BMI≥30 kg/m^2^, with a BMI of 25-30 kg/m^2^ being normal. |
| Lifestyle | Smoke | Recent smoker: People who answer "every day" or "some days" to "Do you now smoke cigarettes" are considered recent smokers. Former smoker: People who have smoked at least 100 cigarettes before, but do not smoke now. Never smoker: People who do not smoke now and have not smoked 100 cigarettes in their lifetime. |
|  | Drinker | It is determined by whether you have drunk at least 12 glasses of alcohol in your life. |
|  | Regular vigorous activity | The assessment on self-reported physical activity have changed since 2007–2010 wave, but the majority of the core questions on physical activity remained unchanged during 2005 and 2010. In 2007-2010, exercise that results in an increase in respiration and heart rate scores for more than 10 minutes per day is considered regular vigorous exercise. In 2006-2016, regular vigorous activity is defined as doing any vigorous activities that caused heavy sweating, or large increases in breathing or heart rate for at least 10 minutes over the past 30 days. |
| Comorbidities | Comorbidities | Comorbidities include thyroid disease, coronary artery disease, high blood pressure, lung problems (asthma, emphysema or chronic bronchitis), chronic liver disease, chronic heart failure, stroke and cancer. |
|  | Diabetes | People who have been told by health care providers to have diabetes, use of hypoglycemic drugs, insulin injections, or related abnormalities (Glycosylated hemoglobin ≥ 6.5% or fasting blood glucose ≥ 126 mg/dL) are considered to have diabetes. |
|  | Depression | A PHQ-9 score of more than 10 is thought to be significantly associated with depressive state. |
|  | Dietary habits | The daily intake of fiber, water, fat, caffeine, total sugar, and total energy. |

**Table S2: Univariate linear regression results of constipation and diarrhea**

|  | P-value | |
| --- | --- | --- |
| characteristic | Diarrhea | Constipation |
| Age |  |  |
| 20-29 vs.70-80  30-39 vs.70-80  40-49 vs.70-80  50-59 vs.70-80  60-69 vs.70-80 | <0.001 | <0.001 |
|  | 0.540 | 0.785 |
|  | 0.666 | 0.549 |
|  | 0.786 | 0.448 |
|  | 0.847 | 0.411 |
| Gender |  |  |
| Female vs. Male | 0.262 | <0.001 |
| Race/Ethnicity |  |  |
| Non-Hispanic Blacks vs. Hispanics | 0.487 | 0.003 |
| Non-Hispanic Whites vs. Hispanics | 0.002 | 0.035 |
| Others vs. Hispanics | 0.109 | 0.852 |
| Education |  |  |
| Below college vs. College and above | 0.001 | <0.001 |
| PIR (income-to-poverty ratio) |  |  |
| Poor vs. Not Poor | 0.012 | <0.001 |
| Smoke |  |  |
| Never smokers vs. Former smokers | 0.023 | 0.001 |
| Recent smokers vs. Former smokers | 0.644 | 0.027 |
| Alcohol |  |  |
| YES vs. NO | 0.358 | <0.001 |
| Regular vigorous activity |  |  |
| YES vs. NO | 0.044 | <0.001 |
| Diabetes |  |  |
| YES vs. NO | <0.001 | 0.053 |
| Body Mass Index |  |  |
| Overweight vs. Normal | <0.001 | 0.024 |
| Obese vs. Normal | 0.002 | 0.001 |
| Comorbidities | <0.001 | 0.465 |
| Depression |  |  |
| YES vs. NO | <0.001 | <0.001 |
| Highest intake of fiber | 0.032 | <0.001 |
| Highest intake of water | 0.002 | <0.001 |
| Highest intake of fat | 0.014 | <0.001 |
| Highest intake of caffeine | 0.819 | <0.001 |
| Highest intake of energy | 0.025 | <0.001 |
| Highest intake of sugar | 0.316 | 0.393 |

**Table S3: Logistic regression results of total n-3PUFA with constipation and diarrhea in sensitive analysis**

|  | OR 95%CI | | | |
| --- | --- | --- | --- | --- |
|  | Q1(0-0.9385g) | Q2(0.9385-1.3825g) | Q3(1.3823-1.9815g) | Q4(1.9815-18.3695g) |
| **Diarrhea** |  |  |  |  |
| Model 1 | Reference | 0.82 [0.65, 1.04] | **0.75 [0.62, 0.91]** | 1.17 [0.96, 1.42] |
| P-value |  | 0.096 | **0.003** | 0.125 |
| Model 2 | Reference | 0.81 [0.65, 1.03] | **0.75 [0.62, 0.90]** | 1.16 [0.95, 1.42] |
| P-value |  | 0.081 | **0.003** | 0.147 |
| Model 3 | Reference | 0.81 [0.64, 1.04] | **0.71 [0.58, 0.88]** | 1.01 [0.76, 1.32] |
| P-value |  | 0.096 | **0.009** | 0.924 |
| **Constipation** |  |  |  |  |
| Model 1 | Reference | 0.86 [0.69, 1.06] | **0.62 [0.49, 0.78]** | **0.42 [0.33, 0.53]** |
| P-value |  | 0.176 | **<0.001** | **<0.001** |
| Model 2 | Reference | 0.87 [0.70, 1.07] | **0.62 [0.49, 0.78]** | **0.43 [0.33, 0.53]** |
| P-value |  | 0.204 | **<0.001** | **<0.001** |
| Model 3 | Reference | 1.01 [0.80, 1.27] | 0.81 [0.62, 1.05] | **0.68 [0.50, 0.92]** |
| P-value |  | 0.947 | 0.101 | **0.014** |
| Model 1: The crude model; Model 2: The age-adjusted model; Model 3: Demographic factors, lifestyle, eating habits and comorbidities were adjusted in this model. | | | | |

**Table S4:** **Logistic regression results of n-3PUFA subtypes with constipation and diarrhea in sensitive analysis**

|  |  |  | | OR 95%CI | | |  |  |  |
| --- | --- | --- | --- | --- | --- | --- | --- | --- | --- |
|  | Model 1 | P-value | Model 2 | | P-value | Model 3 | P-value | Model 4 | P-value |
| **Diarrhea** |  |  |  | |  |  |  |  |  |
| **ALA (g per day)** |  |  |  | |  |  |  |  |  |
| Q1 (0.0000 -0.8645) | Reference |  | Reference | |  | Reference |  | Reference |  |
| Q2 (0.8645-1.2730) | 0.87 [0.68, 1.10] | 0.224 | 0.83 [0.66, 1.04] | | 0.178 | 0.85 [0.68, 1.06] | 0.143 | 0.84 [0.67, 1.06] | 0.131 |
| Q3 (1.2730-1.8075) | **0.75 [0.62, 091]** | **0.004** | **0.74 [0.60, 0.86]** | | **0.004** | **0.71 [0.57, 0.89]** | **0.005** | **0.71 [0.57, 0.89]** | **0.005** |
| Q4 (1.8075-18.3400) | 1.04 [0.81, 1.22] | 0.444 | 1.03 [0.86, 1.24] | | 0.471 | 0.87 [0.71, 1.07] | 0.172 | 0.86 [0.71, 1.04] | 0.114 |
| **DHA (g per day)** |  |  |  | |  |  |  |  |  |
| Q1 (0.0000-0.0105) | Reference |  | Reference | |  | Reference |  | Reference |  |
| Q2 (0.0105-0.0310) | 0.86 [0.71, 1.05] | 0.634 | 0.85 [0.69, 1.03] | | 0.099 | 0.84 [0.68, 1.03] | 0.085 | 0.84 [0.68, 1.03] | 0.086 |
| Q3 (0.0310-0.0720) | 1.04 [0.84, 1.28] | 0.617 | 1.02 [0.82, 1.26] | | 0.865 | 0.98 [0.78, 1.23] | 0.843 | 0.98 [0.78, 1.23] | 0.855 |
| Q4 (0.0720-2.8940) | 1.30 [0.99, 1.69] | 0.117 | 1.27 [0.97, 1.67] | | 0.084 | 1.21 [0.90, 1.64] | 0.192 | 1.21 [0.91, 1.63] | 0.181 |
| **EPA (g per day)** |  |  |  | |  |  |  |  |  |
| Q1 (0.0000-0.0035) | Reference |  | Reference | |  | Reference |  | Reference |  |
| Q2 (0.0035-0.0085) | 0.89 [0.74, 1.07] | 0.204 | 0.88 [0.73, 1.06] | | 0.188 | 0.88 [0.73, 1.05] | 0.152 | 0.87 [0.73, 1.04] | 0.129 |
| Q3 (0.0085-0.0225) | 1.02 [0.84, 1.24] | 0.846 | 1.02 [0.83, 1.24] | | 0.881 | 0.98 [0.78, 1.22] | 0.822 | 0.96 [0.77, 1.20] | 0.733 |
| Q4 (0.0225-1.9910) | 1.13 [0.88, 1.45] | 0.319 | 1.12 [0.87, 1.45] | | 0.355 | 1.10 [0.84, 1.45] | 0.460 | 1.05 [0.83, 1.33] | 0.663 |
| **DPA (g per day)** |  |  |  | |  |  |  |  |  |
| Q1 (0.0000-0.0050) | Reference |  | Reference | |  | Reference |  | Reference |  |
| Q2 (0.0050-0.0130) | 0.83 [0.68, 1.02] | 0.074 | 0.84 [0.68, 1.02] | | 0.080 | 0.83 [0.68, 1.01] | 0.066 | 0.82 [0.68, 1.01] | 0.058 |
| Q3 (0.0130-0.0255) | 0.96 [0.76, 1.22] | 0.759 | 0.97 [0.77, 1.22] | | 0.788 | 0.94 [0.74, 1.19] | 0.608 | 0.93 [0.73, 1.17] | 0.511 |
| Q4 (0.0255-0.4810) | 1.11 [0.86, 1.44] | 0.408 | 1.10 [0.85, 1.43] | | 0.455 | 1.02 [0.75, 1.38] | 0.908 | 0.95 [0.73, 1.25] | 0.721 |
| **Constipation** |  |  |  | |  |  |  |  |  |
| **ALA (g per day)** |  |  |  | |  |  |  |  |  |
| Q1 (0.0000 -0.8645) | Reference |  | Reference | |  | Reference |  | Reference |  |
| Q2 (0.8645-1.2730) | 0.86 [0.70, 1.06] | 0.164 | 0.88 [0.71, 1.07] | | 0.194 | 1.03 [0.84, 1.26] | 0.785 | 1.04 [0.84, 1.28] | 0.711 |
| Q3 (1.2730-1.8075) | **0.64 [0.50, 0.82]** | **0.001** | **0.64 [0.50, 0.82]** | | **0.001** | 0.86 [0.65, 1.13] | 0.169 | 0.87 [0.66, 1.15] | 0.311 |
| Q4 (1.8075-18.3400) | **0.49 [0.39, 0.62]** | **<0.001** | **0.49 [0.39, 0.62]** | | **<0.001** | 0.84 [0.64, 1.11] | 0.206 | 0.86 [0.66, 1.13] | 0.262 |
| **DHA (g per day)** |  |  |  | |  |  |  |  |  |
| Q1 (0.0000-0.0105) | Reference |  | Reference | |  | Reference |  | Reference |  |
| Q2 (0.0105-0.0310) | 0.86 [0.66, 1.11] | 0.229 | 0.87 [0.68, 1.13] | | 0.292 | 0.87 [0.67, 1.13] | 0.295 | 0.86 [0.66, 1.13] | 0.261 |
| Q3 (0.0310-0.0720) | 0.77 [0.59, 0.99] | 0.040 | 0.78 [0.60, 1.01] | | 0.063 | 0.89 [0.68, 1.18] | 0.414 | 0.86 [0.65, 1.16] | 0.306 |
| Q4 (0.0720-2.8940) | **0.49 [0.39, 0.62]** | **<0.001** | **0.50 [0.39, 0.64]** | | **<0.001** | **0.63 [0.49, 0.81]** | **0.001** | **0.62 [0.43, 0.89]** | **0.012** |
| **EPA (g per day)** |  |  |  | |  |  |  |  |  |
| Q1 (0.0000-0.0035) | Reference |  | Reference | |  | Reference |  | Reference |  |
| Q2 (0.0035-0.0085) | 0.85 [0.67, 1.08] | 0.175 | 0.85 [0.67, 1.08] | | 0.181 | 0.88 [0.68, 1.13] | 0.297 | 0.87 [0.67, 1.12] | 0.255 |
| Q3 (0.0085-0.0225) | 0.79 [0.59, 1.05] | 0.102 | 0.79 [0.59, 1.07] | | 0.120 | 0.93 [0.69, 1.25] | 0.625 | 0.91 [0.66, 1.24] | 0.511 |
| Q4 (0.0225-1.9910) | **0.55 [0.44, 0.67]** | **<0.001** | **0.55 [0.45, 0.68]** | | **<0.001** | **0.71 [0.56, 0.89]** | **0.005** | 0.77 [0.58, 1.02] | 0.069 |
| **DPA (g per day)** |  |  |  | |  |  |  |  |  |
| Q1 (0.0000-0.0050) | Reference |  | Reference | |  | Reference |  | Reference |  |
| Q2 (0.0050-0.0130) | 0.98 [0.81, 1.20] | 0.876 | 0.98 [0.81, 1.19] | | 0.843 | 1.01 [0.80, 1.27] | 0.945 | 1.02 [0.82, 1.29] | 0.824 |
| Q3 (0.0130-0.0255) | 0.77 [0.63, 0.94] | 0.012 | 0.77 [0.63, 0.94] | | 0.011 | 0.83 [0.67, 1.03] | 0.091 | 0.86 [0.69, 1.08] | 0.186 |
| Q4 (0.0255-0.4810) | **0.65 [0.52, 0.81]** | **<0.001** | **0.65 [0.52, 0.82]** | | **0.001** | 0.87 [0.68, 1.10] | 0.233 | 0.97 [0.74, 1.28] | 0.826 |
| Model 1: The crude model; Model 2: The age-adjusted model; Model 3: Demographic factors, lifestyle, eating habits and comorbidities were adjusted in this model.  Model 4: Fatty acids were further adjusted on the basis of Model 3. | | | | | | | | | |
